# Mortality outcomes of children and young people who have spent time in care: evidence from Children’s Health in Care in Scotland, a population-wide administrative data cohort study

**DOI:** 10.1136/archdischild-2024-327854

**Published:** 2025-06-05

**Authors:** Denise Brown, Edit Gedeon, Marion Henderson, Alastair H Leyland, Philip Wilson, Mirjam Allik

**Affiliations:** 1 MRC/CSO Social and Public Health Sciences Unit, University of Glasgow, Glasgow, UK; 2 School of Social Work and Social Policy, University of Strathclyde, Glasgow, UK; 3 Institute of Applied Health Sciences, University of Aberdeen, Aberdeen, UK; 4 Institute of Public Health Science, University of Copenhagen, Copenhagen, Denmark

**Keywords:** Child Health, Child Welfare, Epidemiology, Adolescent Health, Mortality

## Abstract

**Objective:**

Individuals who spend time in care during childhood/adolescence face a higher risk of premature mortality compared with those who have not been in care. However, it is unclear whether this risk applies to both external causes of death (eg, accident or suicide) and internal causes (eg, disease), as well as the role of potential confounders.

**Design:**

A descriptive, population-wide cohort study linking administrative data on state school pupils in Scotland with social care records and vital event data for children and young people with (n=12 367) and without (n=6 49 711) experience of care.

**Outcome:**

Premature mortality between 2010 and 2016.

**Analysis:**

Poisson regression, with robust SEs, was used to calculate mortality rate ratios, adjusting for confounders age group, sex, area deprivation, disability, and sociodemographic characteristics at birth.

**Results:**

Of 745 deaths, 58 were among those with care experience. Of these, 66% were due to external causes, compared with 43% in those without care experience. The unadjusted mortality rate was 4.5 (95% CI 2.6 to 7.7) times as high for those with care experience. After adjusting for confounders, the rate ratio (RR) was 3.0 (95% CI 2.0 to 4.4). Adjusting for confounders did not account for differences in external causes; RR 6.5 (95% CI 4.5 to 9.5), but did account for differences in internal causes; RR 1.4 (95% CI 0.9 to 2.3).

**Conclusions:**

Individuals with care experience have higher premature mortality rates, particularly from external causes, compared with those without care experience, a difference not fully accounted for by the confounders considered here. Further research is needed to explore factors contributing to excess external deaths in this population.

**WHAT IS ALREADY KNOWN ON THIS TOPIC**

- Individuals who have spent time in care during childhood/adolescence have a higher mortality risk compared with those without experience of care.
- Limited research exists on the impact of confounders and whether mortality rates are elevated for both external and internal causes of death.

**WHAT THIS STUDY ADDS**

- This research provides a detailed analysis highlighting differences in premature mortality risks between individuals with and without experience of care.
- While the higher risk of death from internal causes can be accounted for by the confounders considered, the risk from external causes remains more than six times as high, even after adjustment.

**HOW THIS STUDY MIGHT AFFECT RESEARCH, PRACTICE OR POLICY**

- This study highlights the elevated risk of death from external causes among individuals with a history of care.
- Further research is needed to explore the underlying factors contributing to this higher risk and guide targeted interventions.

## Introduction

 All children and young people need a stable home and consistent loving relationships to thrive.^1^ Some are placed into care, permanently or temporarily, if their parents cannot provide for them. While the reasons for entering care are varied, many individuals with care experience will have faced considerable trauma and adversity. Such events can lead to poorer outcomes in later life, especially when they occur early and accumulate over time.^2^ While some may benefit from being in care,^3^ many face worse educational outcomes,^4^ social and emotional difficulties,^5^ and poorer health and well-being^6^,^8^ compared with those not in care.

It is widely acknowledged that significant health disparities exist compared with peers who have not grown up in care.^9 10^ This includes higher rates of premature mortality,^11^,^13^ a key marker of overall health and well-being. A recent systematic review and meta-analysis^14^ found that individuals who spent time in care in childhood had more than twice the all-cause mortality risk and over three times the suicide risk in middle age compared with those who had not been in care. These associations persisted even after adjusting for early life factors like parental social class.

Individuals with care experience also face a higher risk of all-cause mortality and suicide in adolescence and young adulthood.^15 16^ Few studies have examined specific causes of death due to the small number of deaths at younger ages. Understanding these patterns is crucial for addressing health inequalities and meeting the needs of children and young people. This requires large population studies comparing mortality outcomes of those with and without care experience. Randomised controlled trials, while considered the gold standard for estimating treatment effects, are rarely feasible in social care settings.^17^ High-quality observational studies using administrative data can help address this gap.

This study uses data from the Children’s Health in Care in Scotland (CHiCS) study, a large population-wide study linking administrative data on state school pupils, social care data, and vital events records, including deaths. We examine all-cause mortality, and deaths from external and internal causes, among children and young people with and without care experience, accounting for age group, sex, area deprivation, disability, and sociodemographic characteristics at birth. External deaths are caused by factors like accidents, suicides or assaults, while internal deaths are those attributable to underlying diseases, such as cancer and neurological conditions.

## Methods

### Population and data

The CHiCS cohort and study design have been described in detail previously.^18^ The cohort includes 13 830 children and young people who were in care between August 2009 and July 2010 (2009/2010), and 649 771 children without care experience. The latter group had no record of being in care from when the Scottish Government’s Children Looked After Statistics (CLAS) were first collected in April 2007 through to the end of the study follow-up in July 2016.

This analysis focuses on children and young people who were included in the 2009 Scottish Government School Pupil Census. Care-experienced children and young people in the CHiCS cohort were those who were in care during 2009/2010. However, some individuals had attended school in 2007 or 2008 but left before 2009, while others did not attend school until 2010 or later. As a result, 1460 care-experienced children and young people were excluded from this analysis. Additionally, one individual without care experience was excluded due to not having a school record in 2009.

The cohort design was specifically established to compare individuals with and without care experience. A third group of children and young people, who were in care between 2007 and 2016 but not during 2009/2010, were not included in the data linkage. This group is estimated to consist of approximately 14 600 individuals, based on the 676 740 pupils in state schools in Scotland in 2009,^19^ of which 662 140 pupils were included in this study.

We also excluded 62 individuals, 3 with care experience and 59 without care experience, who died before the start of mortality follow-up in August 2010. Following these exclusions, the cohort comprised 12 367 children and young people with care experience and 649 711 without care experience. The cohorts were born between 1990 and 2004 and were aged 5–20 years old in August 2010. Mortality outcomes were tracked for 6 years, until July 2016, when the cohorts were aged 11–26 years (see online supplemental figure A1).

The term care-experienced here refers to children and young people (hereafter referred to as young people) who were formally *looked after*, as defined by s17(6) of the Children (Scotland) Act 1995, at some point during 2009/2010, regardless of the duration. This includes young people looked after at home (under a supervision order), in foster care, in residential care or in kinship care (including a small number placed with prospective adopters. Once adopted, young people cease to be looked after). Children looked after at home in Scotland have the same legal status as children looked after away from home and are subject to the same care planning regulations.^20^

Since care-experienced young people may differ from those without care experience with respect to certain characteristics, we controlled for confounding factors related to both care experience and mortality. We adjusted for age group at the start of mortality follow-up (5–9 years, 10–14 years and 15–20 years), sex, deprivation (based on data zone of area of residence at the start of follow-up, grouped into fifths of the population using the Scottish Index of Multiple Deprivation (SIMD) 2009 V.2), and having been assessed as having a disability (yes or no). Both the data zone of area of residence at the start of follow-up and assessed disability were taken from 2009 Pupil Census records. A pupil was deemed disabled if they had a physical or mental impairment with a substantial and long-term (ie, more than a year) adverse effect in their ability to carry out normal day-to-day activities. Disability assessment was performed by a qualified professional (an appropriate health professional, educational psychologist or similar).

Individuals’ own birth records, held by National Records of Scotland (NRS), were available for 11 102 (89.8%) of those with care experience and 571 648 (88.0%) without experience of care. Data zone of area of residence at birth was linked to SIMD 2004, the earliest SIMD release. Birth records also included information on maternal age and parental employment status at birth.

### Outcome

Mortality data were drawn from vital events data held by NRS. Underlying causes of death were coded using the International Classification of Diseases, tenth revision (ICD-10). We examined deaths from all causes, distinguishing between external causes (ICD-10 V01-Y98, F11-16, F18-19) including those related to drug misuse, accidents, assaults and suicide, and internal causes (from across all remaining ICD-10 codes) that were disease-related or illness-related.

### Statistical analysis

We compared age group, sex, deprivation, disability and sociodemographic characteristics at birth of the care-experienced cohort to those without care experience using the χ^2^ test of association. Data were aggregated into 5-year age bands to calculate directly age-standardised mortality rates (ASMRs) per 100 000 person-years for all causes, external causes and internal causes of death, with rates standardised to the European Standard Population 2013. Poisson regression with robust SEs was used to model premature mortality rates, controlling for confounders, with care experience as the exposure. Person-years of follow-up were used as the offset (exposure period). For both external and internal causes of death, we modelled cause-specific mortality rates, with individuals’ time at risk for a specific cause being reduced once they experience death from another cause. All analyses were conducted on the entire cohort and then rerun for the subcohort with linked birth records.

Given the low proportion of missing data, we used complete case analysis to handle missing values. This resulted in the analysis of 12 214 young people with care experience and 649 073 without, representing 98.8% and 99.9% of the starting populations, respectively. Among those with birth records available, 10 946 young people with care experience and 570 083 without care experience were included, corresponding to 98.6% and 99.7% of the starting populations with available birth records, respectively.

## Results

### Cohort characteristics

Table 1 compares cohort characteristics at the start of follow-up, with 1.9% of all young people included in the study having experience of care during 2009/2010. Characteristics significantly differed between those with and without care experience. Young people with care experience were more likely to be male, live in deprived areas, and have a disability.

**Table 1:** Sociodemographic characteristics and disability of young people (from 2009 Pupil Census records)

	All young people (n=662 078)			
	Not care-experienced		Care-experienced	
	N	%	N	%
	649 711	100.0	12 367	100.0
Age group*				
5–9 years	220 451	33.9	4001	32.4
10–14 years	267 729	41.2	5455	44.1
15–20 years	161 531	24.9	2911	23.5
Sex*				
Female	319 413	49.2	5730	46.3
Male	330 298	50.8	6637	53.7
Deprivation fifth*				
Most deprived	140 105	21.6	5545	44.8
Q2	125 751	19.4	2902	23.5
Q3	125 696	19.3	1829	14.8
Q4	129 552	19.9	1250	10.1
Least deprived	127 969	19.7	688	5.6
Missing	638	0.1	153	1.2
Assessed disabled*				
Yes	14 440	2.2	911	7.4
No	635 271	97.8	11 456	92.6

Age group at the start of mortality follow-up in August 2010

* χ2 test of association: p<0.001.

While we could not explore the type of disability through Pupil Census data, since assessed disability was recorded only as yes or no, we were able to examine the main disability recorded in CLAS data for care-experienced young people. We include the breakdowns in online supplemental table A1 as an indicator of the type of disability young people experienced. In CLAS, 12.3% of care-experienced young people had a disability; higher than the rate of 7.4% recorded in the Pupil Census (table 1). Almost two-thirds of care-experienced young people with a disability in CLAS had information about the type of disability; of these, 38.6% had social, emotional and behavioural difficulties, 19.7% had a learning disability, and 16.7% had multiple disabilities.

Online supplemental table A2 provides corresponding output for individuals with linked birth records, with findings consistent with those in table 1. For the group with birth records available, care-experienced individuals were more likely to be born into deprived areas, to younger mothers (under 25 years) and to unemployed parents (table 2).

**Table 2:** Sociodemographic characteristics at birth (1990–2004) of young people

	Young people with birth records available (n=582 750)			
	Not care-experienced		Care-experienced	
	N	%	N	%
	571 648	100.0	11 102	100.0
Deprivation fifth at birth*				
Most deprived	142 241	24.9	6538	58.9
Q2	116 131	20.3	2400	21.6
Q3	107 168	18.7	1266	11.4
Q4	103 601	18.1	619	5.6
Least deprived	101 649	17.8	262	2.4
Missing	858	0.2	17	0.2
Maternal age* (years)				
<20	43 619	7.6	2541	22.9
20–24	105 640	18.5	3673	33.1
25–29	175 098	30.6	2604	23.5
30–34	167 621	29.3	1516	13.7
35–39	68 437	12.0	627	5.6
40+	10 735	1.9	126	1.1
Missing	498	0.1	15	0.1
Parental employment status at birth*				
Employee	405 206	70.9	7040	63.4
Manager	59 903	10.5	201	1.8
Supervisor	17 831	3.1	89	0.8
Self-employed (with employees)	19 993	3.5	100	0.9
Self-employed (without employees)	31 721	5.5	336	3.0
Student/unemployed/not available	36 994	6.5	3336	30.0

* χ2 test of association: p<0.001.

### Mortality

A total of 745 deaths were registered during the follow-up period (August 2010 to July 2016), with 58 deaths occurring among those with care experience and 687 deaths among those without. In care-experienced young people, leading causes included accidental deaths (29%; ICD-10 V01-X59, Y85-86), suicide (26%; ICD-10X60–84, Y10-34, Y87.0, Y87.2), and diseases of the nervous system (10%; ICD-10 G00-99). For those without care experience, common causes were accidental deaths (24%), suicide (17%) and cancer (16%; ICD-10 C00-D48).

The ASMR was 85.1 (95% CI 58.5 to 117.3) per 100 000 person-years for the care-experienced cohort, compared with 14.9 (95% CI 13.6 to 16.3) for those without care experience, a rate 5.7 times as high (table 3). In the care-experienced cohort, 38 (or 66%) deaths were due to external causes compared with 295 (or 43%) in those without care experience. The ASMR for external causes was 54.1 (95% CI 33.4 to 80.4) per 100 000 person-years for the care-experienced cohort, compared with 6.4 (95% CI 5.5 to 7.4) for those without, a rate 8.5 times as high. For internal causes, the ASMR was 31.0 (95% CI 15.6 to 52.5) per 100 000 person-years for the care-experienced cohort, compared with 8.5 (95% CI 7.5 to 9.6) for those without, a rate 3.6 as high. Corresponding rates for those with birth records available are also shown in table 3.

**Table 3:** Age-standardised mortality rates (ASMRs) per 100 000 person-years for all causes, external causes and internal causes of death

	All young people (N=661 287)			
	Not care-experienced (N=649 073)		Care-experienced (N=12 214)	
	N deaths	ASMR (95% CI)	N deaths	ASMR (95% CI)
All deaths	**687**	**14.9 (13.6 to 16.3**)	**58**	**85.1 (58.5 to 117.3**)
External causes	295	6.4 (5.5 to 7.4)	38	54.1 (33.4 to 80.4)
Internal causes	392	8.5 (7.5 to 9.6)	20	31.0 (15.6 to 52.5)

	Young people with birth records (N=581 029)			
	Not care-experienced (N=570 083)		Care-experienced (N=10 946)	
	N deaths	ASMR (95% CI)	N deaths	ASMR (95% CI)
All deaths	**628**	**14.3 (12.9 to 15.6**)	**55**	**85.4 (58.7 to 118.1**)
External causes	270	6.2 (5.3 to 7.2)	37	55.0 (34.2 to 81.8)
Internal causes	358	8.0 (7.1 to 9.1)	18	30.4 (15.0 to 52.2)

**Figure 1:**
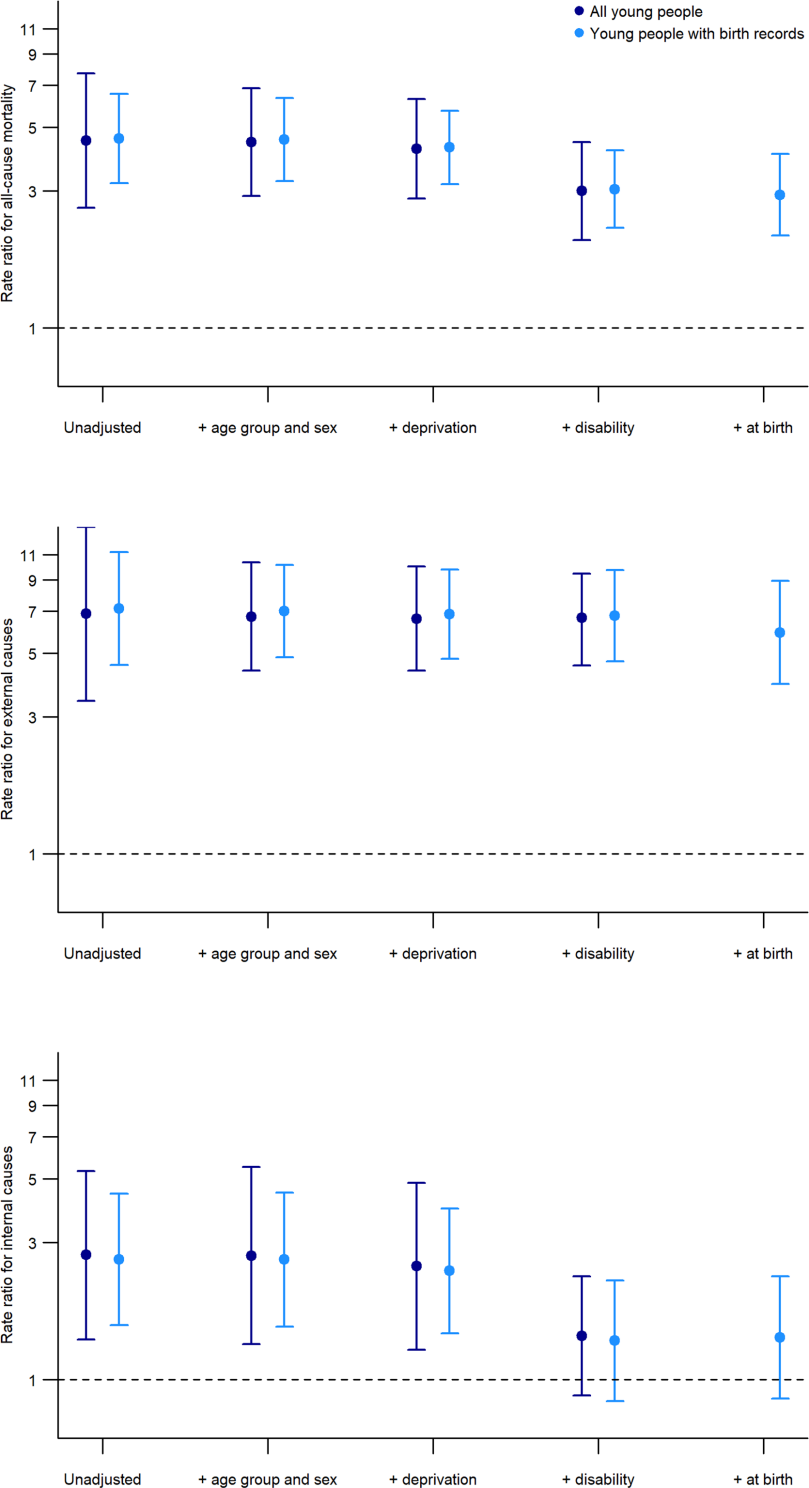
Rate ratios and 95% CIs (log scale) for all-cause mortality (top), external causes (middle) and internal causes (bottom) comparing outcomes for those with care experience to those without care experience. Reference line at 1 indicates no difference between cohorts.

Poisson regression, adjusting for confounders related to both the outcome (mortality) and exposure (experience of care), was used to model the mortality rate of those with care experience compared with those without. Two-way interaction terms (eg, between age group and care experience) were not significant and were therefore not included in the final models. In figure 1 (and online supplemental table A3), we see that the crude mortality rate for all young people was 4.5 (95% CI 2.6 to 7.7) times as high for all young people with care experience compared with those without. Adjusting for age group, sex and deprivation only had a small effect on the adjusted rate ratio. Accounting for disability explained more of the difference in rates; adjusted rate ratio 3.0 (95% CI 2.0 to 4.4). For external causes, the crude rate ratio was 6.9 (95% CI 3.4 to 13.8) and remained 6.5 (95% CI 4.5 to 9.5) after adjustment for age group, sex, deprivation and disability. For internal causes, the crude rate ratio was 2.7 (95% CI 1.4 to 5.3), which attenuated to 2.5 (95% CI 1.3 to 4.8) after adjusting for age group, sex and deprivation. Adjusting for disability accounted for most of the remaining elevated relative risk of death from internal causes, with the rate ratio further reducing to 1.4 (95% CI 0.9 to 2.3).

Rate ratios were similar after adjusting for age group, sex, deprivation and disability among those with available birth records. However, in this group, we were also able to adjust for sociodemographic characteristics at birth (figure 1 and online supplemental table A3). Adjusting for sociodemographic characteristics at birth had minimal effect on the rate ratio for all-cause mortality or internal causes of death. However, it attenuated the rate ratio for external causes from 6.8 (95% CI 4.7 to 9.7) to 5.9 (95% CI 3.9 to 8.9).

## Discussion

All-cause mortality rates were higher among the care-experienced population compared with those without care experience. After adjusting for confounders age group, sex, deprivation and disability for all young people, the risk of death from all causes was 3.0 times as high in the care-experienced cohort. The risk of death from external causes was 6.5 times as high, while the risk from internal causes was not significantly elevated. After adjusting for all confounders for those with birth records available, the risk of death from all causes was 2.9 times as high in the care-experienced cohort, 5.9 times as high for external causes and not significantly elevated for internal causes. These findings are consistent with other studies examining the relationship between care experience and premature mortality. A recent meta-analysis^14^ found a 2.2 times higher risk of all-cause mortality in adulthood for those with a care history, with study-specific estimates varying between 1.04 and 5.8 times. Prior research has also shown increased risk of premature mortality in adolescence, early adulthood^21^,^23^ and midlife,^12 13^ among care-experienced individuals, often due to preventable causes like suicide, substance misuse and accidents, rather than internal causes related to illness or disease.^24^

Adjusting for age group, sex and deprivation had little effect on the relationship between care experience and premature mortality. Despite the strong link between area deprivation and premature mortality,^25^ other factors, not accounted for here, such as neighbourhood or school characteristics, poverty, social relationships, or the timing of the area deprivation measurement may be more important. In line with other studies,^11^ sociodemographic characteristics at birth could not explain the increased relative risk of all-cause mortality among children and young people with experience of care, although they did account for some of the increased relative risk of mortality from external causes. The increased relative risk of mortality from internal causes was explained by accounting for disability. Young people with disabilities are often over-represented in the care-experienced population,^26 27^ with higher prevalence of chronic and neurodevelopmental conditions.^28^

### Strengths and limitations

This study has several strengths. We analysed data from a large population cohort of young people with care experience, comparing them directly to peers without care experience. Individual-level linked administrative data were used, mitigating recall bias. The large cohort size allowed us to examine rare events not possible with smaller samples.

The study has limitations, including potential inaccuracies in recorded information, missing individuals, and reliance on routinely collected data. Factors beyond the care system, such as moving residence or school, could trigger risk behaviours such as illicit drug use.^29^ Young people were considered exposed if they were in care at any point in 2009/2010, regardless of duration, and unexposed if they were never in care between 2007 and 2016 (but could conceivably have been in care before 2007 or after follow-up ended in 2016). The study only includes pupils in state-funded schools (representing just under 96% of all pupils in Scotland in 2009), excluding home-educated or independent school students. We do not include those not yet of school age. While we accounted for disability, given the known increased prevalence among young people in care compared with peers,^26^ disability as measured using the Pupil Census may not fully capture all children with disabilities, particularly those with conditions not readily apparent within a school setting. Some of those with severe disabilities may never have entered the school system. Cohort characteristics are captured at birth and at the start of follow-up. By not including time-varying factors, we may have missed important dynamic effects that could have influenced mortality outcomes over the study period. We examined the underlying cause of death but recognise that in some cases identifying a single cause, whether external or internal, or a combination of both, can be challenging. Finally, we may have underestimated overall mortality. Young people who moved out of Scotland after the start of follow-up and subsequently died would not be included in Scottish death registrations. Similarly, young people who were adopted after follow-up began and later died may not be accurately reflected in death records due to potential name changes making data linkage unfeasible.

### Conclusions and implications

Significant progress has been made in reducing deaths of young people, but not all young people have benefited equally. Young people in families experiencing poverty are more likely to come into contact with the care system.^30^ Often, the care system can provide scaffolding to help young people build their lives, providing stability and creating more positive outcomes. However, care-experienced young people are not a homogeneous group, and some will need more support than others to achieve their potential. The transition from childhood to adolescence and young adulthood is a critical stage where poor mental health, drug use and suicide can drive a steep increase in mortality rates. Although educational success^31^ and aftercare support for young people leaving care^32^ can reduce the risk of premature death, young people with care experience still face higher risks of adversity, even in Nordic countries where young people generally have the highest rates of well-being.^33^

The death of any child is a tragedy, and preventing unnecessary deaths should be a priority. Reviewing the circumstances of a child’s death can provide significant learning and help prevent future deaths.^34^ Care-experienced young people are especially vulnerable to death from external causes and may require additional support for their social, emotional and mental well-being^35^ throughout their time in care and during the transition to independent life. Future research should aim to understand the factors contributing to the higher risk of external causes of death in this population.

## Supplementary material

10.1136/archdischild-2024-327854 online supplemental file 1

## Data Availability

Data may be obtained from a third party and are not publicly available.
