# Supplementary material for: Mortality outcomes of children and young people who have spent time in care: evidence from Children’s Health in Care in Scotland, a population-wide administrative data cohort study
Source: Arch Dis Child. 2025 Jun 5;110(10):e327854. doi: 10.1136/archdischild-2024-327854 (PMC12505078; doi:10.1136/archdischild-2024-327854)
Supplement: online supplemental file 1 [file archdischild-110-10-s001.pdf]

Supplementary appendix

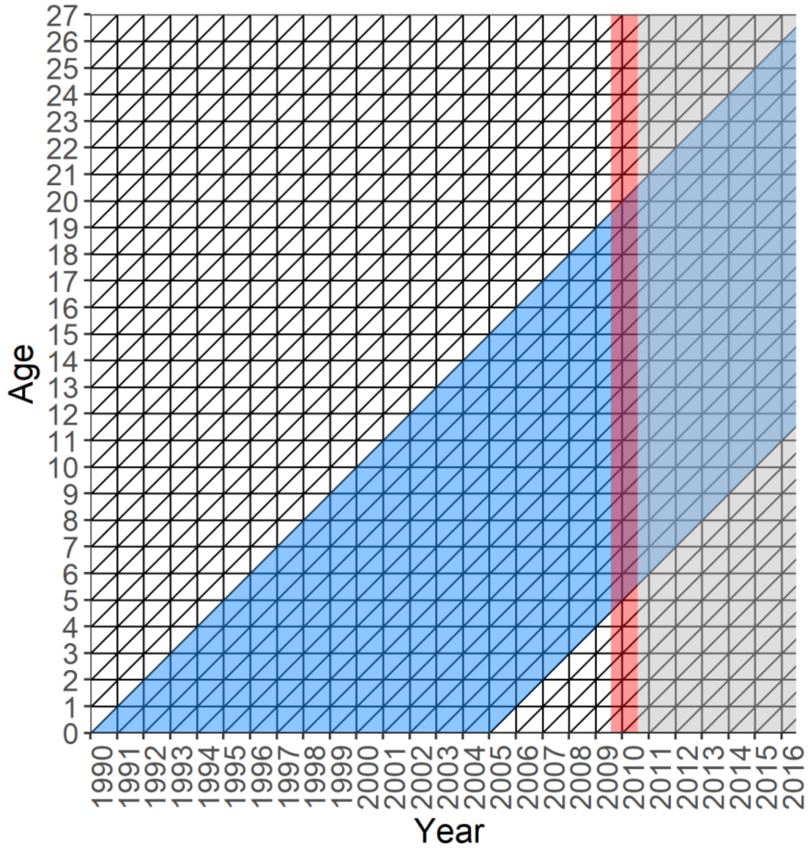

Figure A1: Lexis diagram showing cohorts born 1990-2004 (blue), exposure August 2009 - July 2010 (red) and mortality follow-up period August 2010 - July 2016 (grey).

Table A1: Main disability of care experienced young people (from Children Looked After Statistics (CLAS)).

	**Care experienced young people (N=12,367)**	
	N	%
No disability	*10,851*	*87.7*
Has a disability	*1,516*	*12.3*
**Main disability** (where known*)	**939**	**100.0**
Social, emotional, and behavioural difficulty	362	38.6
Learning disability	185	19.7
Multiple disabilities	157	16.7
Other chronic illness/disability	74	7.9
Autistic spectrum disorder	56	6.0
Physical or motor impairment	38	4.0
Visual or hearing impairment	29	3.1
Mental health problem	24	2.6
Language and communication disorder	14	1.5

*Main disability is unknown for 577 young people. CLAS recorded information on type of disability prior to 2015/16. Where records have been linked annually to create a longitudinal dataset, and include 2015/16 or 2016/17 extracts, disability is given only as yes or no.

Table A2: Sociodemographic characteristics and disability of young people (from 2009 Pupil Census records. Age group at the start of mortality follow-up in August 2010). For those with birth records available.

	**Young people with birth records (N=582,750)**			
	**Not care experienced**		**Care experienced**	
	**N**	**%**	**N**	**%**
	**571,648**	**100.0**	**11,102**	**100.0**
**Age group[a]**				
5-9 years	195,584	34.2	3,653	32.9
10-14 years	235,949	41.3	4,904	44.2
15-20 years	140,115	24.5	2,545	22.9
**Sex[a]**				
Female	281,199	49.2	5,139	46.3
Male	290,449	50.8	5,963	53.7
**Deprivation fifth[a]**				
Most deprived	123,318	21.6	5,024	45.3
Q2	112,752	19.7	2,627	23.7
Q3	110,150	19.3	1,622	14.6
Q4	112,366	19.7	1,100	9.9
Least deprived	112,837	19.7	605	5.4
Missing	*225*	*0.0*	*124*	*1.1*
**Assessed disabled[a]**				
Yes	12,687	2.2	827	7.4
No	558,961	97.8	10,275	92.6

[a] χ^2 test of association: p<0.001

Table A3: Rate ratios (95% confidence intervals) for all-cause mortality, and external and internal causes of death, comparing individuals with and without care experience. Results are presented for all young people (top rows) and separately for those with linked birth records (bottom rows). Corresponds to rates shown in Figure 1.

	M1: Unadjusted	M2: M1 + age group and sex	M3: M2 + deprivation	M4: M3 + disability	M5: M4 + at birth
**All young people (N=661,287)**					
**All deaths**					
Not care experienced	*1.00 (ref)*	*1.00 (ref)*	*1.00 (ref)*	*1.00 (ref)*	
Care experienced	4.49 (2.62, 7.71)	4.44 (2.88, 6.85)	4.20 (2.82, 6.26)	3.00 (2.02, 4.44)	
**External causes**					
Not care experienced	*1.00 (ref)*	*1.00 (ref)*	*1.00 (ref)*	*1.00 (ref)*	
Care experienced	6.86 (3.41, 13.78)	6.71 (4.35, 10.35)	6.60 (4.34, 10.04)	6.54 (4.53, 9.45)	
**Internal causes**					
Not care experienced	*1.00 (ref)*	*1.00 (ref)*	*1.00 (ref)*	*1.00 (ref)*	
Care experienced	2.72 (1.38, 5.32)	2.70 (1.33, 5.50)	2.48 (1.27, 4.84)	1.42 (0.88, 2.29)	
**Young people with birth records (N=581,029)**					
**All deaths**					
Not care experienced	*1.00 (ref)*	*1.00 (ref)*	*1.00 (ref)*	*1.00 (ref)*	*1.00 (ref)*
Care experienced	4.57 (3.19, 6.54)	4.53 (3.24, 6.32)	4.26 (3.17, 5.71)	3.04 (2.23, 4.15)	2.91 (2.10, 4.03)
**External causes**					
Not care experienced	*1.00 (ref)*	*1.00 (ref)*	*1.00 (ref)*	*1.00 (ref)*	*1.00 (ref)*
Care experienced	7.15 (4.54, 11.27)	7.02 (4.84, 10.17)	6.84 (4.77, 9.80)	6.76 (4.69, 9.73)	5.90 (3.90, 8.92)
**Internal causes**					
Not care experienced	*1.00 (ref)*	*1.00 (ref)*	*1.00 (ref)*	*1.00 (ref)*	*1.00 (ref)*
Care experienced	2.62 (1.55, 4.43)	2.62 (1.53, 4.47)	2.39 (1.45, 3.94)	1.37 (0.84, 2.21)	1.40 (0.86, 2.29)
